# Integrating Tobacco Dependence Treatment and Tobacco-Free Standards Into Addiction Treatment: New Jersey’s Experience

**Published:** 2006

**Authors:** Jonathan Foulds, Jill Williams, Bernice Order-Connors, Nancy Edwards, Martha Dwyer, Anna Kline, Douglas M. Ziedonis

**Affiliations:** Jonathan Foulds, Ph.D., is associate professor and director at the Tobacco Dependence Program, School of Public Health, University of Medicine and Dentistry of New Jersey (UMDNJ), New Brunswick, New Jersey and clinical associate professor at the Department of Psychiatry, Robert Wood Johnson Medical School, UMDNJ, New Brunswick, New Jersey. Jill Williams, M.D., is associate professor at both the Department of Psychiatry, Robert Wood Johnson Medical School, UMDNJ, New Brunswick, New Jersey, and at the Tobacco Dependence Program, School of Public Health, UMDNJ, New Brunswick, New Jersey. Bernice Order-Connors, L.C.S.W., is an instructor and Nancy Edwards, L.C.A.D.C., is training and education coordinator, both at the Tobacco Dependence Program, School of Public Health, UMDNJ, New Brunswick, New Jersey. Martha Dwyer is Choices program director and Anna Kline, Ph.D., is a research fellow, both at the Department of Psychiatry, Robert Wood Johnson Medical School, UMDNJ, New Brunswick, New Jersey. Douglas M. Ziedonis, M.D., M.P.H., is a professor and director in the Division of Addiction Psychiatry, Tobacco Dependence Program, Robert Wood Johnson Medical School, UMDNJ, New Brunswick, New Jersey

Tobacco dependence is a serious and deadly problem for patients in treatment for alcohol and other drug (AOD) dependence. Such patients have increased mortality rates compared with the general population, and more than half die from tobacco-caused illnesses (Hurt et al. 1996). The majority of patients seeking treatment for substance use disorders state that cigarettes would be at least as hard or harder to quit compared with their primary problem substance (Kozlowski et al. 1989). Despite clear evidence of tobacco addiction, and major tobacco-caused health consequences among substance users, tobacco use traditionally has been minimized or ignored as an issue in addictions treatment settings. For example, AOD treatment facilities in the United States routinely ban alcohol and illicit drug use and drug dealing on their grounds; however, fewer than 1 in 10 ban tobacco use (Richter et al. 2005). These systems issues, in addition to biological, psychological, and other social factors, have resulted in extremely high tobacco use among patients in treatment for substance use disorders in the United States (70 to 95 percent), whereas smoking prevalence in the general population has fallen to less than 21 percent (CDC 2005).

New Jersey was the first State to require that all residential addiction treatment programs assess and treat patients for tobacco dependence and maintain tobacco-free facilities (including grounds). An evaluation of this policy change found that tobacco dependence treatment can be successfully integrated into residential substance abuse treatment programs through policy regulation, training, and the provision of nicotine replacement therapy (NRT) (Williams et al. 2005). Many other addiction treatment agencies (both residential and outpatient) around the country now have implemented or are planning to implement similar policies to ensure that their patients receive appropriate assessment and treatment of their tobacco dependence while receiving treatment for addiction to other substances. This paper aims to summarize the lessons learned from the experience in New Jersey.

## History

Numerous agencies and individuals were involved in the preliminary work that led to the New Jersey policy change. Starting in 1991, the late Professor John Slade led a project funded by the Robert Wood Johnson Foundation called, “Addressing Tobacco in the Treatment of Other Addictions.” This project trained New Jersey’s addiction providers in tobacco treatment and provided the rationale that tobacco should be treated on par with other addictive substances in these settings. Many treatment providers were influenced by the project, and the Division of Addiction Services at the New Jersey State Department of Health and Senior Services provided additional funding. During the mid-1990s, addiction providers, the Division of Addiction Services, and individuals from the “Addressing Tobacco” project discussed the integration of tobacco into the division’s licensure standards. In 1999 the State of New Jersey passed licensure standards that required residential addiction treatment providers to assess and treat patients for tobacco dependence and maintain tobacco-free grounds at all residential treatment sites (with this later requirement phased in by November of 2001). By 2000 the Division was receiving funding for tobacco control from New Jersey’s Comprehensive Tobacco Control Programs; some of this funding provided training and free NRT for residential addiction treatment providers to help implement the standards. The Tobacco Dependence Program at the University of Medicine and Dentistry of New Jersey (UMDNJ) School of Public Health administered the training and NRT.

The key ingredients for policy development and implementation in New Jersey were (1) a committed leader to “champion” this issue, (2) initial “buy-in” training to convince treatment providers that treating tobacco is the right thing to do, (3) willingness on the part of the State Division of Addiction Services to include the policy within the licensure standards for providers, (4) funding for training and NRT, and (5) availability of expertise in tobacco treatment and training.

## Implementation and Results

Members of the addictions treatment community initially were concerned that clients in New Jersey would refuse to come to tobacco-free addiction treatment programs or be negatively impacted in some way by the policy. Staff members were concerned that the introduction of tobacco dependence treatment would possibly disrupt the treatment milieu and that the change to tobacco-free grounds would result in an increase in premature or irregular discharges from residential addictions treatment. To increase effective implementation, extensive training was provided on tobacco assessment and treatment for both management and front-line staff, and free NRT (in the form of nicotine patch and gum) was provided to all agencies for patient use (and later also for staff ). New Jersey’s Division of Addiction Services made an early decision to monitor the implementation and to enforce the new regulations through encouragement only. Usual disciplinary actions such as issuing a citation or revoking a license were not enacted for a failure to comply with the policy.

**Figure:**
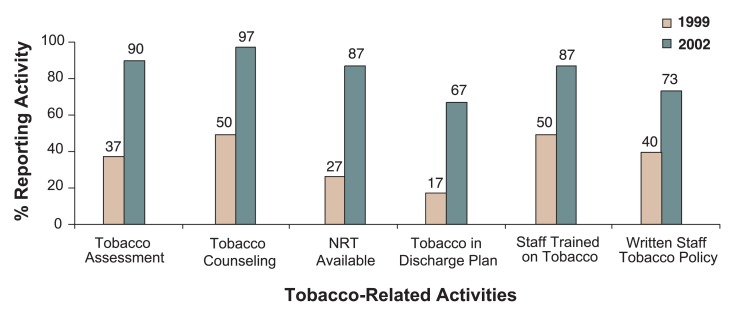
Percentage of New Jersey residential addiction treatment agencies reporting tobacco-related activities before (1999) and after (2002) Statewide Tobacco Licensure Standards (*n* = 30).

Williams and colleagues (2005) evaluated this policy change process using a study design consisting of observation before and after the policy change, with no comparison group in all 33 residential treatment programs in New Jersey. The main client measures of interest were smoking status, attitudes about the tobacco regulations, willingness to stop tobacco use, acceptance and utilization of NRT, and length of stay in residential addictions treatment. The main program and staff outcomes were the provision of tobacco dependence treatment and tobacco-free grounds, and the distribution of NRT, as well as qualitative feedback of their impressions and attitudes about the tobacco provisions.

**Table:** Representative Qualitative Comments From Directors of New Jersey’s Residential Addiction Treatment Facilities After Implementation of Tobacco-Free Treatment Standards.

Questions	Response
What do you believe has been the most beneficial aspect of the Tobacco and Nicotine Provisions?	“Acknowledgement of nicotine dependence and addressing it as part of client and staff addiction.” “It has raised consciousness that this is a killing addiction and increased awareness of tobacco-caused illnesses.” “Opportunity to experience benefits of a tobacco-free life.” “Tobacco-free policy supports those who are trying to quit.” “Clients and staff stopping or cutting back [their tobacco use].” “Increased self-esteem by showing they can do it.” “Smoke breaks [no longer] interrupt treatment.” “Prompted systematic review of tobacco policies and procedures.”
What do you believe has been the most problematic aspect of the Tobacco and Nicotine Provisions?	“Lack of enforcement by the State has marginalized financially facilities that went tobacco-free.” “Lack of a level playing field.” “Fear of reduced admissions and decreased revenues.” “Making cultural change during initial transition.” “Challenge of developing policies and procedures that integrate tobacco and ensur- ing it is followed.” “Residents not willing to quit smoking; not seeing it as a problem.” “Staff resistance to tobacco-free grounds.” “Smoking staff not providing consistent message.”
What practice or technique have you found to be of the greatest value in successfully integrating tobacco depen- dence treatment into the usual practice at your facility?	“Creating a context suggesting that tobacco is abnormal; not normal in society at large.” “Took steps to prepare and set date.” “The message is this is an addiction and we treat addiction.” “Starting it at admission and continuing it through the entire process.” “Nicotine replacement is key.” “Staff that have quit smoking are a real benefit.” “The practice of not having staff that smoke or smell [of smoke].” “Stages of Readiness for Change Model and motivational interviewing.” “Raising awareness and giving incentives for clean time” “UMDNJ Tobacco Dependence Program’s trainings, services, and materials.”
If it were up to me, this is how I would see tobacco addressed in residential substance abuse treatment programs.	“What is outlined in the Standards now. Tobacco fully integrated and addressed, just like other drugs.” “State should enforce [the] Standards. There are no consequences for noncompli- ance.” “Education, education. Working with Readiness to Change Model. Raise cognitive dissonance.” “More intensive treatment for clients requesting it.” “Mandatory treatment throughout [the] State, with NRT provided for clients and staff, with increased educational trainings.”

The policy implementation was associated with a large increase in the quantity and quality of tobacco dependence treatment in residential addictions programs, even though only 50 percent of facilities were fully compliant with the tobacco-free grounds requirement. Staff training was well attended across the State, and tobacco assessment, treatment planning, and treatment of tobacco dependence (including use of NRT) all substantially increased from the period before the tobacco licensure standards were implemented (1999) to the period after full implementation (2002). The Figure shows the percentage of programs carrying out various tobacco-related activities before and after the policy change. Rates of premature discharges were not different between smokers and nonsmokers, and there was no increase in irregular discharges or reduction in the proportion of smokers among those entering residential treatment compared with prior years (Williams et al. 2005). Two-thirds of smokers interviewed at admission expressed a desire to stop or cut down on their tobacco use, and at discharge almost half thought that the tobacco-free policy had helped them address their tobacco use.

A survey of the executive and clinical directors of 30 New Jersey residential programs in 2003 also provided some useful qualitative feedback on the implementation process. A representative selection of comments from those interviews is provided in the Table.

These comments indicate that the program directors recognized the benefits of treating tobacco in addictions treatment and of creating an environment that supports such treatment. Some of the comments in the Table reflect the fact that although the New Jersey licensure standards were intended to mandate tobacco treatment and tobacco-free grounds, in practice the lack of strict enforcement by the State resulted in a situation in which programs were able to choose whether to maintain a strict tobacco-free grounds policy. This led to a perception that programs with tobacco-free grounds would suffer reduced referrals and admissions. The lack of a “level playing field” regarding implementation and enforcement of tobacco-free grounds was a source of concern in the survey participants. Despite these issues, the survey results suggest that the tobacco-free grounds requirement was an important catalyst for organizational change in programs implementing tobacco treatment policies and practices.

In New Jersey the tobacco-free campus policy was implemented after the requirement for assessment and treatment. This was partly to give agencies more time to prepare for what was perceived as the most challenging component and to reduce initial resistance when the standards were announced. In other States it may not be necessary to separate the two components, but providers will likely require some time to train staff and to adequately prepare for going tobacco free (e.g., around 6 months from the time the policy is announced). Similarly, we would recommend that residential and outpatient addiction treatment services integrate tobacco treatment and policies simultaneously, so as to better provide continuity of tobacco treatment provision. Both nicotine patches and nicotine gum were made available in most treatment programs, but the nicotine patch was far more popular. This partly was because many residential programs prohibited any use of gum and partly because the patch was more convenient for clinical staff to administer and monitor as a “one-a-day” treatment. The patch also has the advantage that unlike the nonnicotine medications, it does not require a physician’s prescription and does not take a week or more to be effective.

## Discussion and Lessons Learned

Since the initial implementation and evaluation project, the interest in providing tobacco dependence treatment as part of addictions treatment in New Jersey has continued, despite the lack of enforcement of the tobacco-free grounds component of the licensure standards. Tobacco dependence treatment and NRT became available to staff and at outpatient facilities. Some large behavioral health and addiction treatment facilities in New Jersey (e.g., Ann Klein Forensic Hospital and Princeton House) that are not technically subject to the licensure standards also have voluntarily chosen to implement similar policies including tobacco-free grounds, staff training, formulary changes to enhance treatment options, and routine implementation of tobacco treatment.

The main lessons from the New Jersey experience (Williams et al. 2005) are the following:

1. Tobacco dependence treatment can be fully integrated into addiction treatment programs.
2. Most patients in addiction treatment programs want to change their tobacco use.
3. Treating tobacco dependence in the context of tobacco-free grounds does not lead to patients leaving treatment early.
4. The greatest resistance to implementing a tobacco-free policy typically comes from staff rather than patients (with staff who smoke but are in recovery from other addictions sometimes feeling that their sobriety is being challenged).
5. Thorough staff preparation and training, along with availability of NRT (for both staff and patients who smoke), are important components of implementation.
6. Implementation of tobacco-free grounds is the most challenging aspect of the policy but also is an important driver of other organizational changes (e.g., policies for staff tobacco use, availability of NRT, etc.).
7. Not enforcing tobacco-free policies can detract from their effectiveness.

An increasing number of individual agencies and whole-State treatment systems around the country are coming to terms with the compelling rationale for treating tobacco dependence on par with alcohol use disorders in the context of addiction treatment programs. It is not a small or easy cultural shift to transform from an addiction treatment agency that largely ignores or condones tobacco use to one that assesses and treats tobacco use and dependence on par with alcohol use and dependence. However, the experience in New Jersey suggests that combining policy change, staff training, and additional treatment resources can successfully achieve the transformation. We also have been working with providers in other States (e.g., New York, Ohio, and Massachusetts) who are now addressing tobacco in addictions treatment on a Statewide basis. We have found that addiction treatment providers who initially were resistant to such changes become comfortable with the idea that “drug free is tobacco free” and “tobacco dependence is an addiction and we treat addiction.”
